# Cortical thickness of the inferior parietal lobule as a potential predictor of relapse in men with alcohol dependence

**DOI:** 10.21203/rs.3.rs-2628081/v1

**Published:** 2023-03-07

**Authors:** Kebing Yang, Ruonan Du, Qingyan Yang, Rongjiang Zhao, Fengmei Fan, Song Chen, Xingguang Luo, Shuping Tan, Zhiren Wang, Ting Yu, Baopeng Tian, Thang M. Le, Chiang-Shan R. Li, Yunlong Tan

**Affiliations:** Peking University HuiLongGuan Clinical Medical School, Beijing Huilongguan Hospital; Peking University HuiLongGuan Clinical Medical School, Beijing Huilongguan Hospital; Peking University HuiLongGuan Clinical Medical School, Beijing Huilongguan Hospital; Peking University HuiLongGuan Clinical Medical School, Beijing Huilongguan Hospital; Peking University HuiLongGuan Clinical Medical School, Beijing Huilongguan Hospital; Peking University HuiLongGuan Clinical Medical School, Beijing Huilongguan Hospital; Yale University School of Medicine; Peking University HuiLongGuan Clinical Medical School, Beijing Huilongguan Hospital; Peking University HuiLongGuan Clinical Medical School, Beijing Huilongguan Hospital; Peking University HuiLongGuan Clinical Medical School, Beijing Huilongguan Hospital; Peking University HuiLongGuan Clinical Medical School, Beijing Huilongguan Hospital; Yale University School of Medicine; Yale University School of Medicine; Peking University HuiLongGuan Clinical Medical School, Beijing Huilongguan Hospital

**Keywords:** Alcohol dependence, Cortical thickness, Inferior parietal lobule, Relapse, Predictor

## Abstract

**Background:**

Alcohol dependence (AD) is a disorder with a high recurrence rate that leads to a considerable public health burden. The risk of relapse appears to be related to a complex interplay of multiple factors. Herein, we aimed to explore the potential neural predictors of relapse in Chinese male patients with AD.

**Methods:**

This study enrolled 58 male patients with AD who had undergone acute detoxification. General demographic information and clinical features were collected. Magnetic resonance imaging (MRI) data were used to measure cortical thickness across 34 regions of the brain. Patients were followed up at 6 months, and 51 patients completed the follow-up visit. These patients were divided into a relapser and an abstainer group. A binary logistic regression analysis was performed to investigate the potential risk factors of relapse.

**Results:**

Compared to abstainers, relapsers showed higher inattention and non-planning impulsivity on the 11th version of the Barratt Impulsive Scale. The cortical thicknesses of the inferior-parietal lobule were significantly greater in abstainers compared with those in relapsers. Furthermore, binary logistic regression analysis showed that the thickness of the inferior parietal lobule predicted relapse.

**Conclusions:**

Relapsers show poorer impulse control than abstainers, and MRI imaging shows a decreased thickness of the inferior parietal lobule in relapsers. Our results indicate the thickness of the inferior parietal lobule as a potential relapse predictor in male patients with AD.

## Introduction

Alcohol dependence (AD) is a chronic, relapsing illness, and the World Health Organization (WHO) has highlighted AD as a major public health issue worldwide(Organization 2018). According to a recent survey on the prevalence of mental disorders in China, the lifetime prevalence of AD was reported to be 1.3%(Xiang et al. 2022), lower than the worldwide rate(Steel et al. 2014), since only the data for AD, not alcohol use disorder (AUD) was surveyed with the structured clinical interview for the 4th edition of the Diagnostic Statistical Manual of Mental Disorders (DSM-IV).

Cumulative evidence has shown that the recurrence rate of AD is about 60% within 6 months after acute detoxification(Terra et al. 2008). A complex interplay of multiple factors, including alcohol craving(Snelleman et al. 2018), poor impulse control(Czapla et al. 2016), and negative affect(Paulino et al. 2017)ADDIN, contributes to relapse. In particular, the effects of chronic alcohol consumption on the brain may aggravate these pathophysiological processes and dispose individuals to relapse(Durazzo et al. 2011).

Alcohol craving can be characterized as an emotional response in which an individual is motivated to seek out and consume alcohol(Le et al. 2020). One line of evidence suggests that a higher level of craving at the time of hospitalization is associated with relapse following residential addiction treatment(Oslin et al. 2009). Other studies also support the role of craving in relapse and the utility of evaluating craving with tools such as the visual analog scale (VAS) and alcohol urge questionnaire (AUQ) in assessing relapse risk(Iwanicka and Olajossy 2015; Schneekloth et al. 2012). Furthermore, negative emotional states conduce to cue-elicited craving, particularly in individuals who drink to alleviate depression and anxiety(Pombo et al. 2016). Since Lee and colleagues(Lee et al. 2013) found that the activation of the inferior parietal lobule (IPL) and dorsolateral prefrontal cortex (DLPFC) decreased, and limbic region increased along with increased craving responsivity in patients with AD, the prefrontal-IPL-limbic circuit could be identified as an underlying neural mechanism of emotional involvement in pathological alcohol craving.

Extensive research suggests that AD is associated with impairments of impulse control and goal-directed behavior(Bailey et al. 2014), which was correlated with relapse(Batschelet et al. 2021; Schmidt et al. 2017). On 18F-fluorodeoxyglucose positron emission tomography (FDG-PET), patients with AD had presented widespread lower metabolic rates in the dorsolateral and nearly all orbital prefrontal cortex and IPL compared with healthy controls, with most marked decreases in frontal areas, consistent with diminished impulse control(Bralet et al. 2022). To clarify impaired impulse control, the delay discounting task (DDT) was frequently tested in patients with AD. During delay stimulus presentations of a previous study(Dennis et al. 2020), patients with AD exhibited a greater propensity for impulsive choices to steep discounting patterns (i.e., preferring smaller, immediate rather than larger, delayed rewards) and lower activation compared to healthy controls, notably in the anterior cingulate cortex (ACC), DLPFC, and IPL. In line with these findings, another study suggested that in DDT, the process of action representation in the IPL may play an important part in the larger parieto-frontal activity responsible for movement selection(Bečev et al. 2021).

Consistent the aforementioned findings, neuroimaging studies involving alcohol craving and impaired impulse control associated with the relapse risk of AD have gradually focused on the prefrontal and parietal cortex. During the early abstinence period in AD, morphological abnormalities in the frontal and parietal regions have been observed in patients who ultimately relapse following treatment(Morley et al. 2020). Numerous studies have found that abstinence from alcohol addiction is actually a complex process of goal-directed behavior that integrates higher-order cognitive functions and volitional activities(Azuar et al. 2021; Garbusow et al. 2014; Powell et al. 2022). The parietal cortex in humans comprises somatosensory areas and several higher-order functions associated with the executive control network(Whitlock 2017). Previous findings have indicated a key contribution of the IPL in this process(Desmurget and Sirigu 2009; Haggard 2008). Since goal-directed actions were considered to be triggered by the motor intention of wanting to move, which is under the control of the inferior parietal regions and specifies a general goal to be reached before planning, we propose that IPL might mediate the early subjective experience of wanting to abstain from alcohol(Desmurget and Sirigu 2012). Although the evidence presented above suggests that the structure and function of the frontal and parietal cortex, especially the IPL, may be essential to maintain long-term abstinence and reduce relapse, the functional correlates of altered cortical thickness and whether the parietal cortical thickness may serve as a structural brain marker of relapse in AD remain unclear.

To address this gap in research, the present study aimed to expand upon our earlier work(Yang et al. 2020) with modestly larger sample size and an approximately 6-month follow-up to determine if frontal and/or parietal lobe thickness predicts relapse within 180 days in a group of Chinese patients with AD. We also aimed to determine whether a relationship exists between the cortical thickness of regions of interest (ROIs) and demographic, clinical, and psychological variables meanwhile.

## Materials And Methods

### Participants

**Figure 1:**
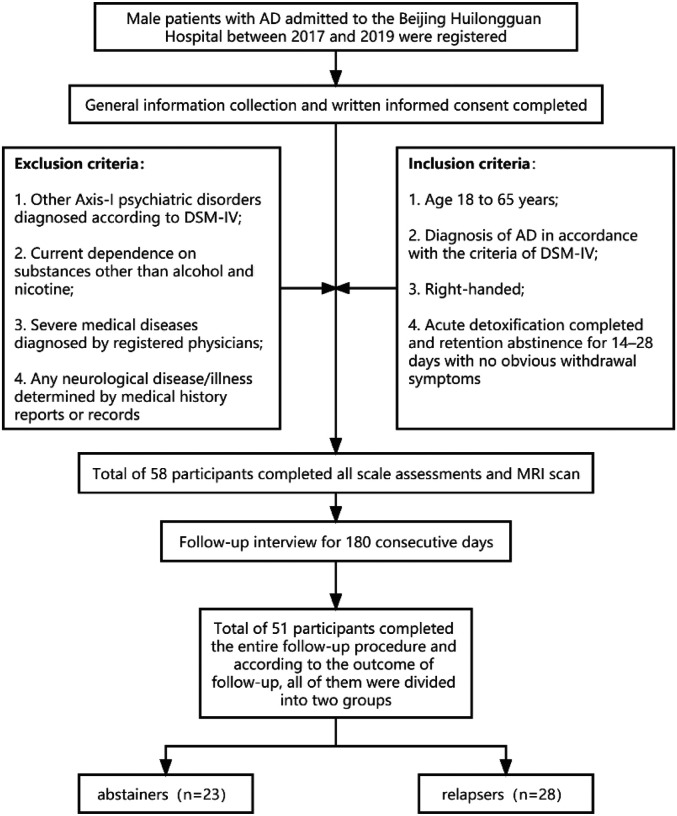
Flow-chart of the study design

This study enrolled 58 male patients with AD, all of Han Chinese descent, and had been admitted to Beijing Huilongguan Hospital between 2017 and 2019. All the AD inpatients recruited adhered to the inclusion criteria as follows: (a) age 18 to 65 years; (b) diagnosis of AD in accordance with the criteria of the DSM-IV(Association 2000); (c) right-handed; (d) acute detoxification completed and retention abstinence for 14–28 days with no obvious withdrawal symptoms and a Clinical Institute Withdrawal Assessment for Alcohol (CIWA)(Sullivan et al. 1989) score < 3. Patients with (a) other Axis-I psychiatric disorders diagnosed by professional psychiatrists according to DSM-IV (e.g., schizophrenia, obsessive-compulsive disorder, bipolar disorder, major depression, etc.), (b) current dependence on substances other than alcohol and nicotine (e.g., benzodiazepines, cannabis, etc.), (c) severe medical diseases diagnosed by registered physicians according to systematic examination (e.g., unstable hypertension or diabetes, myocardial infarction, liver cirrhosis, etc.), or (d) any neurological disease/illness determined by medical history reports or records were excluded. Prior to study inclusion, all participants provided written informed consent in agreement with the Declaration of Helsinki. The study was approved by the ethics committee of Beijing Huilongguan Hospital. The flow-chart of the study design, including the decision tree for recruitment, is shown in Fig. 1.

### General information collection

General information, including age, education, occupation, and smoker status, was documented before admission to the hospital. A semi-structured questionnaire was used to record the conditions of lifetime alcohol consumption(Sobell et al. 1996), including age at first drink, age at onset, total duration of AD, the need for morning alcohol consumption, and family history of alcoholism. Meanwhile, if a transient state characterized by decreased clarity of consciousness and disorientation or seizure-like manifestations such as loss of consciousness and convulsions of the limbs presented after abrupt cessation or reduction of alcohol consumption so far, we determined them as delirium or seizure for patients with AD and vice versa. Since the inpatients enrolled in this study were all daily drinkers, the Quantity-Frequency (QF methods) assessment(Sobell et al. 1982) was only used to determine the mean number of daily drinks (10 grams of ethanol per standard drink) over the last 3 months before detoxification.

### Scale Assessments at baseline

In the study, the baseline was defined as the period of acute detoxification completed and retention abstinence for 14–28 days with no obvious withdrawal symptoms and CIWA score < 3. All scales were evaluated at the baseline before magnetic resonance scanning as follows:

1. The Chinese version of the Alcohol Use Disorder Identification Test (AUDIT)(Saunders et al. 1993), which consists of 10 questions, was applied to assess the severity of alcohol consumption, as validated previously(Li et al. 2011). Total AUDIT score ranges from 0 to 40, with a higher score indicating a more severe drinking problem and a higher risk of AD.
2. A validated Chinese version of the 11th version of the Barratt Impulsive Scale (BIS-11) was used to evaluate impulsivity(Huang et al. 2013). The full BIS-11 scale has 30 items and consists of three dimensions: inattention impulsivity, motor impulsivity, and non-planning impulsivity. A higher score represents poorer impulse control. Visual Analogue Scale (VAS) is a sensitive and comparable method for alcohol craving assessment. It is specific to draw a 10 cm line on the paper, “0” at one end, indicating no craving for drinking; “10” on the other, indicating the most severe craving for drinking; the middle part means different degrees of craving. Let the patients mark the cross line to indicate the extent of the craving(Zhu et al. 2019).
3. The Alcohol Urge Questionnaire (AUQ)(Bohn et al. 1995) was used to measure the current urgency of drinking during withdrawal. This scale is one of the most important tools used to measure craving, both in clinical trials and therapeutic practice(Iwanicka and Olajossy 2015). Total AUQ scores between 8–56, with greater values indicating a greater urge to drink alcohol.
4. Self-assessment scales of anxiety and depression (SAS and SDS) were administered(Zung 1971). Both scales consist of 20 items, with a higher score indicating more severe symptoms of anxiety and depression.

### Follow-up assessment

All patients were encouraged to remain abstinent and scheduled for a follow-up interview. A follow-up questionnaire was used to assess post-treatment outcomes, including whether the patient had relapsed or not (first re-drink will be considered as relapse), time to relapse (number of abstinence days), and the quantity and frequency of alcohol consumption after relapse(Tao YJ et al. 2019). The outcome of relapse was defined as returning to drinking; that is, relapse can be viewed as “not completely abstinent” in our study. The follow-up period was accurately 180 days, beginning from the time of patient discharge back to the real world. Those who had completely abstained from alcohol for more than or equal to 180 days were considered abstainers, and the time to first re-drink was regarded as 180 days by default, whereas those failed to maintain abstinence for 180 consecutive days were considered relapsers, and their time to first re-drink was accurately recorded (day as the unit of time). Follow-up interviews involved face-to-face and/or telephone contact with family members living with the patient during the 1st to 5th day of every month for approximately 6 months. If a patient missed an appointment for a face-to-face interview or could not conduct a phone interview three consecutive times, he was considered lost to follow-up. A total of 51 patients with AD completed the entire follow-up procedure.

### Magnetic resonance imaging

After acute detoxification, all patients with AD underwent magnetic resonance imaging (MRI). MRI data were acquired using a Siemens 3T MRI scanner. Head motion was minimized using foam pads. Whole-brain structural MRI was acquired with a sagittal 3D-magnetisation-prepared rapid acquisition gradient echo sequence using the following parameters: echo time (TE) = 2.22 ms, inversion time (TI) = 1,000 ms, repetition time (TR) = 2,400 ms, flip angle (FA) = 8°, field-of-view (FOV) = 240 mm × 256 mm, matrix size = 256 mm × 240 mm, and thickness/gap = 0.8/0 mm.

### Analysis of structural MRI data

Regional measures of cortical thickness (mm) for 34 cortical regions of interest (ROIs) according to Desikan-Kiliany Atlas(Jao et al. 2021) and total grey matter volume (TGV) were obtained using FreeSurfer(Fischl 2012) (http://surfer.nmr.mgh.harvard.edu). TGV was used as a covariate in all analyses to account for differences in head size. For quality control, we followed the ENIGMA pipeline (http://enigma.ini.usc.edu/): all ROIs with a thickness > 1.5 or < 1.5 times the interquartile range were identified and visualized by overlaying the segmented on the patients’ anatomical images. Only ROI data for which segmentation was judged to be accurate upon visual inspection were subjected to statistical analyses. The present study combined ROIs across both hemispheres.

### Statistical analysis

Chi-square (χ^2^) tests and independent samples t-tests were used to analyze the categorical (e.g., employment status) and continuous data (e.g., age and years of education), respectively. The quantitative data that do not conform to the normal distribution (e.g., time to first re-drink) were described by the median (upper and lower quartiles) and analyzed using non-parametric tests. The level of statistical significance was set at P < 0.05 (two-tailed).

Thickness values for the 34 cortical ROIs (right- and left-hemispheric regions combined) were compared between relapsers and abstainers over the 180-day follow-up period using univariate linear regression analyses, where the cortical thickness of each ROI was used as the dependent variable, and group (relapsers/abstainers), age, smoking status, education-years, and TGV were entered as predictors. The threshold for statistical significance was set at p < 0.001470588 (i.e., 0.05/34) to correct for multiple comparisons (Bonferroni correction).

Zero-order Spearman correlation analyses were conducted to evaluate whether the first time to re-drink significantly correlated with several important clinical categorical variables in the relapsers group, e.g., whether the individual experienced delirium over the last 3 months or not (scored 1 or 0 respectively). Zero-order Pearson correlation analyses were conducted to assess the associations of the cortical thickness of differential brain regions with BIS-11 inattention, motor and non-planning scores, and VAS, AUQ, and AUDIT total scores. For all of these analyses, significance was considered at *P* < 0.05.

After identification of the cortical ROI that differed significantly between relapsers and abstainers, we performed a binary logistic regression model constructed using a stepwise method (F-to-enter probability ≤ 0.05 and F-to-remove ≥ 0.10). Occurrence of relapse during the 180-day follow-up period was regarded as the dependent variable (0 = abstainers; 1 = relapsers), while the application of clinical features for binary logistic regression and the thickness of inferior-parietal lobule were used as independent variables with age, education-years, smoking status, and TGV as covariates, so that the effects of these factors on relapse could be controlled for. All statistical analyses were performed using SPSS 20.0 (IBM Corp., Armonk, NY, USA).

## Results

### Demographic and clinical characteristics

Based on whether relapse had occurred, we divided all 51 patients who completed the entire follow-up into a relapser (n = 28) and an abstainer (n = 23) group. We observed no significant group differences in age (t=-1.00, *P* = 0.32), years of education (t=-0.91, *P* = 0.37), age at onset of illness (t=-2.04, P = 0.05), duration of illness (t = 1.55, P = 0.13), or the number of mean daily drinks over the last 3 months (t = 1.08, *P* = 0.29). There were no significant group differences in smoking status, employment status, drinking in the morning, family history of alcoholism, presence of delirium, or seizures (all P > 0.05). Table 1 shows the data.

Compared to abstainers, relapsers scored significantly higher on BIS-11 impulsivity, particularly in the inattention (t = 2.60, *P* = 0.01) and non-planning (t = 2.60, *P* = 0.01) subscore. At baseline, no other clinical characteristics showed significant group differences (Table 2).

### Cortical thickness and the relationship with clinical characteristics

The cortical thickness of the inferior and superior parietal lobule and supramarginal gyrus were higher in abstainers compared with that in relapsers after accounting for age, years of education, smoking status, and TGV (all *P*’s < 0.05). Only the cortical thickness of the inferior parietal lobule showed significant group differences after Bonferroni correction (*P* < 0.05/34) (Table 3). In relapsers (n = 28), the variable of time to first re-drink showed a negative correlation with seizure (*r*=−0.47, *P* = 0.012) and delirium (*r*=−0.45, *P* = 0.017) experienced during 3 months after withdrawal recently (Table 4). Additionally, the zero-order Pearson analysis revealed significant negative correlations between BIS inattention score and cortical thickness of supramarginal gyrus (*r*=−0.35, *P* = 0.013) and between AUQ total score and cortical thickness of IPL and supramarginal gyrus (*r*=−0.31 and − 0.33, *P* = 0.026 and 0.017) (Table 5).

### Binary logistic regression with controls for confounding factors

**Figure 2:**
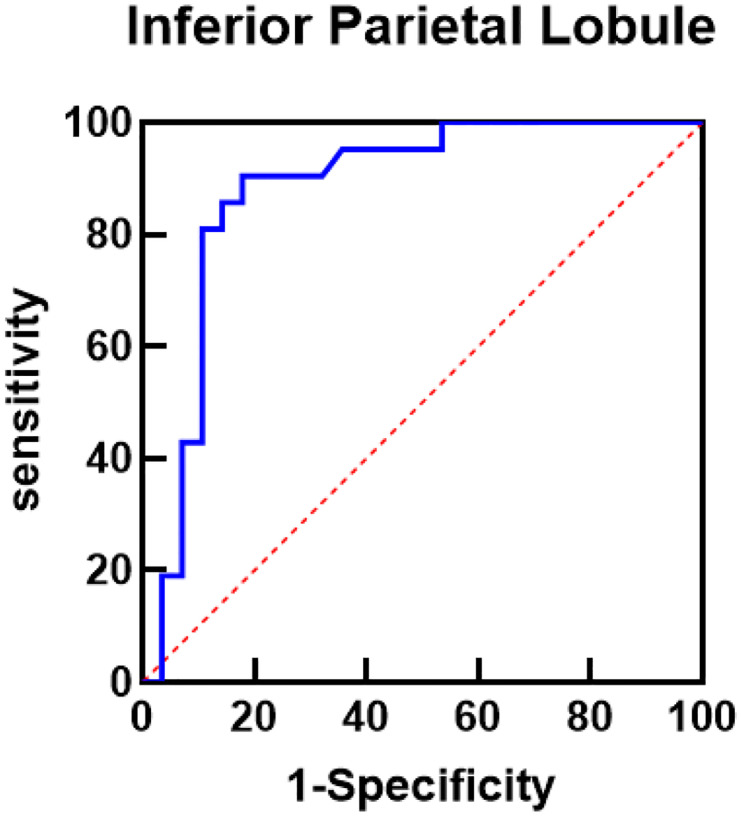
ROC curve analysis of inferior parietal lobule (IPL) predicting relapse within 180 days. Note: Area under the curve (AUC) is 0.878, 95% *CI*: 0.775–0.982, *P* value < 0.001.

After age, education-years, smoking status, age at onset, AUQ total score, inattention, motor and non-planning of BIS-11, TGV, and the thickness of inferior parietal lobule put into a monofactor analysis, variables with P < 0.1 in the results were used as independent variables. Occurrence of relapse during follow-up period was used as the dependent variable (0 = abstainers; 1 = relapsers). Then the binary logistic regression model was performed with forward stepwise regression. The results showed that the thickness of the inferior parietal lobule was a risk factor (*β*=−10.940, *P* = 0.001, *95% CI*: 0.000–0.012) of relapse. Additionally, a lower non-planning score was a protective factor (*β* = 0.214, *P* = 0.030, 95% *CI:* 1.021–1.503) for relapse. The ROC curve results showed that the area under the ROC curve of the final logistic regression model was 0.878 (*95% CI:* 0.775–0.982, *P* < 0.01), as shown in Fig. 2.

## Discussion

We identified cortical thickness of the inferior parietal lobule as the most significant predictor of relapse in Chinese male patients with AD. Age, education, smoking status, employment, age of onset, duration of illness, drinking in the morning, family history of alcoholism, mean daily drinks, and the presence of delirium or seizures did not influence the likelihood of relapse. Although in the relapsers group of our study, the variables significantly associated with the time to first re-drink were seizures and delirium, that is, the time to first re-drink was shorter in patients with seizures and delirium experienced within 3 months after withdrawal, there was no significant difference in the two variables between the relapser and abstainer groups. A recent study has also found the contribution of neurotoxicity of alcohol withdrawal syndrome to structural brain alterations. Even if the patients who experienced seizures and/or delirium tremens during acute withdrawal were not included in the study, the severity of such brain impairment may be correlated with the prognosis of patients with AD(Laniepce et al. 2020). Moreover, in another study, it was suggested that younger age at first drink and the presence of delirium during acute withdrawal predicted more severe alcohol cravings(Kaya et al. 2021). The results of our study did not support the opinion of relapse to drinking associated with clinically significant impairment (e.g., age at first drink, drinking in the morning, family history of alcoholism, presence of delirium or seizures, and mean daily drinks), except for the age of onset. The difference in results may be attributed to the enrollment of severe AD inpatients and adoption of different metrics and procedures in our study. Nevertheless, our study found a minor difference in age of onset between abstainers and relapsers, suggesting that younger age of onset may be a risk factor for relapse within 6 months. Although this variable was not successfully incorporated into the prediction model in the subsequent modeling, we should continue to pay attention to the age of onset since this point is consistent with most findings in previous studies(Moure-Rodríguez and Caamano-Isorna 2020; Rial et al. 2020; Zhu et al. 2017).

Cortical thickness is related to the number or density of cells in a column of the cerebral cortex, which is subject to the neurotoxic effects of chronic alcohol consumption. Cortical thickness was chosen as the primary measure in this study since previous work has indicated the index to be more sensitive to group differences than grey matter volume in imaging studies(Winkler et al. 2010). One study reported that focal areas of increased cortical thickness were observed in the brains of healthy male controls compared to alcoholic males(Momenan et al. 2012), and according to another recent report, cerebral cortex injury was most pronounced in the frontal and parietal cortical regions and associated with reduced glucose metabolism in patients with AD(Tomasi et al. 2019). A diminished parietal cortex may impair the allocation of attention and impulse control during high-order cognitive function(Wang et al. 2019). Another study likewise suggested that patients with AD had lower metabolic rates in the frontal, temporal, and parietal lobes compared to healthy volunteers(Bralet et al. 2022) since these brain regions were mainly involved in the rewarding neural circuit and executive control network, which are critical in inhibitory control and maintaining sobriety(Dennis et al. 2020; Gladwin et al. 2013). Additionally, craving is thought to be an important mechanism involved in the development and maintenance of AD(Koob and Volkow 2010). Impulsivity, craving, and brain damage mentioned above can be considered potential predictors of relapse in AD(Czapla et al. 2016; Durazzo and Meyerhoff 2017). In particular, along with our findings that the thickness of the IPL was most significantly decreased in relapsers than abstainers, we also demonstrated higher inattention and non-planning impulsivity in relapsers relative to abstainers, consistent with an earlier study(Czapla et al. 2016) and other work suggesting impulsive decision-making as a hallmark characteristic of AD(Dennis et al. 2020). In our study, we did not clarify the relationship between impulsive control impairment of BIS-11 and cortical thickness decrease of IPL in all AD inpatients enrolled in our study. However, if the data were not treated strictly according to the Bonferroni correction, we could also see that inattention of impulsivity was negatively correlated with the thickness of the supramarginal gyrus cortex (one of the ROIs before the Bonferroni correction), and AUQ total score was negatively associated with the cortical thickness of IPL and supramarginal gyrus. Even if the severity of alcohol craving represented by AUQ scores did not reveal the significant difference between relapsers and abstainers, the correlation between severe craving and decreased cortical thickness of ROIs is still worthy of further attention. In fact, these findings are similar to previous studies(Kose et al. 2015; Seo and Sinha 2014; Zhu et al. 2017).

Therefore, the differences in cortical thickness between abstainers and relapsers, broadly consistent with earlier studies(Durazzo et al. 2011; Rando et al. 2011), suggest the utility of the macrostructural measures in identifying patients at risk of relapse(Durazzo and Meyerhoff 2017). Similar to the results of our previous study in AD relative to controls(Yang et al. 2020), the current finding indicates inferior parietal cortical thickness as a critical marker of alcoholism. By virtue of several higher-order functions mediated by the posterior parietal cortex, different portions of the posterior parietal cortex participate in multiple cognitive processes, including sensorimotor integration, spatial attention, decision-making, working memory, early motor planning, as well as more complex behaviors(Witkiewitz 2011). As we all know, decreased cortical thickness in the anterior cingulate, frontal and parietal regions of the executive control network might explain the maintenance of addictions in terms of an impaired ‘reflective’ system, enhancing and maintaining the salience of potential punishments in working memory(Gianelli et al. 2022). According to the findings of our study, the decreased inferior parietal cortices and higher non-planning impulsivity are implicated as the risk factors of relapse in male patients with AD. Actually, keeping abstinence from alcohol dependence is a key high-order cognitive function that the IPL involves as an adaptive task-control hub of the fronto-parietal control network(Cole et al. 2013). In other words, deficient internal initiation of behavior mediated by the IPL may be sufficient to reduce goal-directed behavior and then lead to relapse for patients with AD(Martínez-Vázquez and Gail 2018; Tumati et al. 2019). Unfortunately, our study did not find significant differences in the frontal lobe, another important brain functional region in this neural network. More studies with a larger sample size would be needed to investigate the potential inter-relationship between impulse control, parietal cortical structure and function, and alcohol addiction.

### Limitations

There were limitations to the present study. Firstly, the modest sample size may limit the generalizability of our findings. Future discovery research on AD must be undertaken with larger samples using prospective observational methods(Stout 2021). Secondly, our definition of relapse (i.e., any alcohol consumption) was based on the consideration that any level of alcohol resumption was associated with a poorer psychosocial function(Durazzo and Meyerhoff 2017). Other relapse metrics should be thoroughly investigated to reflect the severity of relapse in future work. Thirdly, we did not examine the influence of sex on AD relapse as we only included male patients with AD. A considerable literature suggests important sex-dependent differences in the pathophysiological processes of alcoholism(Rossetti et al. 2021). Again, more studies are needed to address this issue. Fourthly, we did not assess coping skills, stress, self-efficacy, marital status, and social support, which have all been shown to influence and/or predict drinking behavior after treatment(Le et al. 2021). Lastly, we focused on cortical thickness. Other morphometric measures including gray matter volumes and surface represent potential neural markers of AD(Chye et al. 2020).

## Conclusion

Male patients with AD who were relapsers after acute detoxification showed more inattentional and non-planning impulsivity than abstainers. Moreover, MRI results demonstrated that the thickness of the inferior parietal lobule predicted relapse. These findings add to the literature regarding neural markers of alcoholism.

## Figures and Tables

**Table 1:** Demographic and general clinical information of relapsers and abstainers: mean(SD).

	Relapsers (n = 28)	Abstainers (n = 23)	*t*/*χ*^2^/*Z*-value	*p*-value
Age, years	43.86(8.51)	46.48(10.15)	−1.00	0.32
Education, years	11.50(3.48)	12.35(3.13)	−0.91	0.37
employed(yes/not, Y/N)^a^	17/11	16/7	0.43	0.51
smoking status (Y/N) ^a^	23/5	20/3	0.22	0.64
Age at first drink, years	18.96(4.08)	20.35(4.89)	−1.10	0.28
Age of onset, years	31.68(7.00)	37.17(11.23)	−2.04	0.05
Duration of illness, years	12.25(8.77)	9.35(4.22)	1.55	0.13
Drinking in the morning(Y/N)^a^	24/4	20/3	0.02	0.90
family history of alcoholism(Y/N)^a^	5/23	2/21	0.90	0.34
delirium(Y/N)^a^	9/19	4/19	1.45	0.23
seizure(Y/N)^a^	4/24	4/19	0.09	0.76
Mean daily drinks ^b^	20.97(7.96)	18.85(5.55)	1.08	0.29
Time to first re-drink, days ^c^	30(14, 57)	180(180, 180)	−6.40	0.00

a Chi-square (χ^2^) test: smoking status (Y/N) = Y: current or previous smoker, N: never smoker; employed (Y/N) = Y: employed, N: unemployed; Drinking in the morning, Family history of alcoholism, Delirum and Seizure (Y/N) = Y: experienced within 3 months recently, N: not experienced.

b One drink contains 10 g of pure alcohol.

c Described by the median (upper and lower quartiles) and analyzed by the Mann-Whitney test.

**Table 2:** Testing results of all clinical scales between relapsers and abstainers: mean(SD).

	Relapsers (n = 28)	Abstainers (n = 23)	t-value	p-value
AUDIT total score	29.43(5.69)	28.83(5.33)	0.39	0.70
BIS-11 inattention	14.61(2.97)	12.52(2.52)	2.60	0.01
BIS-11 motor	21.04(1.45)	19.74(3.29)	1.75	0.09
BIS-11 nonplaning	29.89(5.11)	26.52(3.93)	2.60	0.01
VAS	3.43(2.03)	2.43(2.56)	1.55	0.13
AUQ total score	28.79(9.52)	24.09(8.65)	1.83	0.07
SAS	26.64(3.95)	25.96(2.82)	0.70	0.49
SDS	28.39(4.71)	27.65(5.18)	0.54	0.60

Independent sample *t* test. *P* values < 0.05 mean significant difference between two groups. AUDIT, the Alcohol Use Disorder Identification Test; BIS-11, the 11th version of the Barratt Impulsive Scale; VAS, Visual Analogue Scale; AUQ, Alcohol Urge Questionnaire; SAS, Self-assessment scales of anxiety; SDS, Self-assessment scales of depression.

**Table 3:** The cortical thickness of all brain regions between relapsers and abstainers: mean(SD).

Cortical thickness (mm)	Relapsers (n = 28)	Abstainers (n = 23)	F-value	P-value
Banks of superior temporal sulcus	2.50(0.17)	2.55(0.12)	1.17	0.286
Caudal anterior cingulate cortex	2.71(0.21)	2.58(0.23)	1.65	0.206
Caudal middle frontal gyrus	2.50(0.18)	2.53(0.12)	0.01	0.909
Cuneus cortex	1.90(0.14)	1.95(0.17)	0.00	0.987
Entorhinal cortex	3.49(0.28)	3.55(0.35)	0.62	0.435
Fusiform gyrus	2.80(0.1 5)	2.83(0.10)	0.23	0.636
**Inferior parietal lobule**	**2.23(0.16)**	**2.43(0.11)**	**15.84**	**0.000**
Inferior temporal gyrus	2.79(0.22)	2.88(0.14)	1.96	0.168
Isthmus cingulate cortex	2.28(0.21)	2.24(0.1 5)	0.65	0.424
Lateral occipital cortex	2.03(0.1 5)	2.12(0.14)	3.24	0.078
Lateral orbitofrontal gyrus	2.73(0.23)	2.73(0.1 5)	0.02	0.895
Lingual gyrus	2.10(0.15)	2.15(0.11)	0.30	0.587
Medial orbitofrontal gyrus	2.57(0.22)	2.62(0.1 5)	0.34	0.564
Middle temporal gyrus	2.79(0.21)	2.87(0.17)	1.97	0.168
Parahippocampal gyrus	2.51(0.27)	2.51(0.21)	0.00	0.958
Paracentral lobule	2.41(0.21)	2.44(0.17)	0.12	0.730
Pars opercularis	2.52(0.19)	2.58(0.09)	0.27	0.606
Pars orbitalis	2.66(0.24)	2.70(0.14)	0.27	0.605
Pars triangularis	2.42(0.18)	2.45(0.13)	0.04	0.836
Pericalcarine cortex	1.63(0.13)	1.73(0.18)	2.76	0.104
Postcentral gyrus	2.00(0.20)	2.08(0.17)	0.44	0.509
Posterior cingulate cortex	2.44(0.14)	2.46(0.13)	0.90	0.348
Precentral gyrus	2.50(0.20)	2.57(0.16)	1.27	0.266
Precuneus	2.31(0.18)	2.36(0.13)	0.70	0.406
Rostral anterior cingulate cortex	2.88(0.22)	2.80(0.13)	1.08	0.305
Rostral middle frontal gyrus	2.33(0.18)	2.36(0.09)	0.08	0.773
Superior frontal gyrus	2.67(0.17)	2.70(0.12)	0.06	0.806
Superior parietal lobule	1.95(0.16)	2.07(0.1 5)	4.92	0.032
Superior temporal gyrus	2.72(0.24)	2.79(0.1 5)	0.74	0.394
Supramarginal gyrus	2.34(0.14)	2.49(0.13)	9.32	0.004
Frontal pole	2.75(0.27)	2.72(0.19)	0.02	0.893
Temporal pole	3.65(0.36)	3.72(0.33)	1.15	0.289
Transverse temporal gyrus	2.34(0.21)	2.39(0.21)	0.52	0.473
Insula	2.97(0.23)	2.92(0.20)	0.08	0.781

One-way ANOVA. Significant *P* values < 0.001470588 after the Bonferroni correction are presented in bold.

**Table 4:** Zero-order Spearman correlations across the time to first re-drink and clinical categorical variables in relapsers.

	1	2	3	4	5
1. Drinking in the morning(Y/N)					
2. Family history of alcoholism(Y/N)	−0.076				
3. Delirium(Y/N)	0.281	−0.121			
4. Seizure(Y/N)	0.167	−0.190	0.375*		
5. Time to first re-drink, days	−0.196	0.197	−0.446*	−0.469*	

* Significant at *P* < 0.05.

**Table 5:** Zero-order Pearson correlations across the cortical thickness of differential brain regions and clinical variables.

	1	2	3	4	5	6	7	8	9
1. Inferior parietal lobule									
2. Superior parietal lobule	0.739**								
3. Supramarginal gyrus	0.761**	0.745**							
4. BIS inattention	−0.227	−0.222	−0.345*						
5. BIS motor	−0.127	−0.095	−0.162	0.507**					
6. BIS non-planing	−0.201	−0.229	−0.269	0.533**	0.338*				
7. AUQ	−0.312*	−0.1 53	−0.332*	0.235	0.046	0.208			
8. AUDIT	0.149	−0.020	−0.090	0.287*	0.186	0.385**	0.248		
9. VAS	0.019	0.092	−0.012	0.237	0.122	0.214	0.214	0.351*	

AUDIT, the Alcohol Use Disorder Identification Test; BIS-11, the 11th version of the Barratt Impulsive Scale; VAS, Visual Analogue Scale; AUQ, Alcohol Urge Questionnaire.

* Significant at *P* < 0.05.

** Significant at *P* < 0.01.

## Data Availability

The data underlying this article are available in the article and in its online supplementary material.
